# Editorial: Emerging Mechanisms for Skeletal Muscle Mass Regulation

**DOI:** 10.3389/fcell.2021.764095

**Published:** 2021-09-27

**Authors:** Yuji Ogura, Shuichi Sato, Yann S. Gallot, Susan Tsivitse Arthur

**Affiliations:** ^1^Department of Physiology, St. Marianna University School of Medicine, Kawasaki, Japan; ^2^School of Kinesiology, University of Louisiana at Lafayette, Lafayette, LA, United States; ^3^LBEPS, Univ Evry, IRBA, Université Paris Saclay, Evry, France; ^4^Department of Applied Physiology, Health and Clinical Sciences University of North Carolina at Charlotte, Charlotte, NC, United States

**Keywords:** myocyte, signaling, cancer, homeostasis, myogenesis

Skeletal muscle is considered an essential tissue involved in many physiological functions such as metabolism, thermoregulation, respiration, and locomotion. It has become clear that genetic and environmental factors are intricately involved in regulating skeletal muscle volume or myofiber size. The complexity of the orchestration of the signaling pathways that regulate these factors is intriguing. Muscle biologists strive to elucidate the myriad of signaling interactions critical for muscle volume and homeostasis. In this topic, we aimed to gain new insights into the basis of muscle volumetric and homeostatic regulation. We collected nine papers that embody novel or important concepts to skeletal myology. The three main areas compose the manuscripts: adult myogenesis, skeletal muscle mass regulation, and cancer-induced muscle weakness.

Adult myogenesis is a critical process to establish proper myofiber. The first article examined the role of hypoxia-inducible factor 1α (HIF-1α) in myogenesis by Cirillo et al. The authors have previously reported that hypoxia-preconditioning upregulated both HIF-1α and Wnt7a followed by enhanced hypertrophy (Cirillo et al., 2017). In this study, the authors showed that HIF-1α directly binds to the Wnt7a promotor region and stimulates its expression under hypoxic conditions. Activation of HIF-1α is sufficient to improve muscle regeneration via activation of Wnt7a signaling in mice. These results provide novel evidence of the cooperation of HIF-1α and Wnt7a in myogenic machinery.

The unfolded protein response (UPR), an evolutionarily conserved intracellular signaling mechanism, is an important event involved in adult myogenesis (Bohnert et al., 2018). The article by Tan et al. demonstrates that the knockdown of RNA-dependent protein kinase-like ER eukaryotic translation initiation factor 2 alpha kinase (PERK), one of the three UPR signaling branches, exaggerated myotube formation through perturbing micro RNA (miRNA) networks in the immortalized mouse skeletal myoblast cell line C2C12 culture. While *in vivo* confirmation is required, the results propose the unique relationship between UPR and miRNA in the context of myogenic differentiation.

Myod family inhibitor (Mdfi or I-mfa) is a myogenic repressor (Chen et al., 1996). However, the inhibitory role of Mdfi is controversial. Using CRISPR/Cas9-engineered Mdfi-overexpressing C2C12 cells, Huang et al. found that Mdfi promoted several myogenic regulatory factors and fast-to-slow transition of myosin heavy chain (MyHC) isoforms expression. Thus, the results suggest that Mdfi plays a positive role in myogenic differentiation under a culture setting.

Requirements volumetric muscle gain with resistance training is a practical research area with universal implications. Using genetically-modified mice, Joanne et al. report in their original research study that a lack of desmin, an intermediate filament protein of skeletal muscle, caused disruption of sarcomere integrity and mitochondrial abnormalities. Four weeks of mechanical overload revealed a functional decline in overloaded plantaris muscle in the desmin-knockout mice, associated with a disrupted autophagy system. These data demonstrated that desmin is necessary for overload-induced muscle adaptation.

This Research Topic includes two comprehensive review articles discussing skeletal muscle homeostasis. White provides a compelling discussion on the relationship of pathways for muscle protein synthesis and metabolism including how metabolic signaling such as AMP-activated protein kinase (AMPK) affects anabolism and catabolism processes including mechanistic target of rapamycin (mTOR) signaling. Considerable effort has focused on the role of branched-chain amino acids (BCAAs) trafficking in coordinating protein turnover.

The second review article discusses transduction of mechanical signals to the nucleus. This research area has recently received attention in skeletal myology. A mechanical network in the nucleoskeleton and cytoskeleton is a crucial element to understand cellular mechanobiology. Proper mechanotransduction to the nucleus can initiate appropriate response and adaptation of muscle cells to local environmental changes. van Ingen and Kirby summarized the current concept and topic of nuclear mechanobiology related to skeletal muscle homeostasis and function.

Cancer biology is now an important field of skeletal myology because cancer development causes substantial volumetric reduction of skeletal muscle in patients. There are three articles in this collection that examine the crosstalk of skeletal muscle with cancer. Straughn et al. demonstrated that the anti-tumorigenic compound Withaferin A (WFA), purified from the plant *Withania somnifera*, directly acts to the skeletal muscle to induce hypertrophic adaptation under ovarian cancer environment. Thus, WFA could be a potential therapeutic means to ameliorate both ovarian cancer and cancer-related muscle wasting.

Colon-26 (C26) tumor cells-derived factor inhibits myotube growth signals. Since passive stretch has been identified to increase protein synthesis and muscle volume, Halle et al. demonstrated that passive stretch in C2C12 myotubes could prevent diminished protein synthesis signaling and fast-type myosin expression induced by C26 tumor-derived factors in a cultured setting. This study provides initial evidence that mechanical stimulus could counteract cancer-induced muscle deconditioning.

Finally, Mallard et al. reviewed the research to understand the alterations in skeletal muscle in breast cancer patients, particularly as a consequence of chemotherapy, and then prospectively described potential mechanisms based on multiple types of cancer-related conditions. This review article also includes information regarding the fundamental signaling pathways that regulate skeletal muscle volume.

We hope that our Research Topic is informative to advance the field of skeletal muscle research. Lastly, we thank the authors for their contributions to this collection.

## Author Contributions

YO wrote the first draft of the manuscript. YO, SS, YSG, and STA wrote the manuscript. All authors contributed to manuscript revision, read, and approved the submitted version.

## Funding

SS was supported by Louisiana Board of Regents Support Fund (LEQSF (2017-20)-RD-A-22).

## Conflict of Interest

The authors declare that the research was conducted in the absence of any commercial or financial relationships that could be construed as a potential conflict of interest.

## Publisher's Note

All claims expressed in this article are solely those of the authors and do not necessarily represent those of their affiliated organizations, or those of the publisher, the editors and the reviewers. Any product that may be evaluated in this article, or claim that may be made by its manufacturer, is not guaranteed or endorsed by the publisher.
